# Characterization of simultaneous uptake of xylose and glucose in *Caldicellulosiruptor kronotskyensis* for optimal hydrogen production

**DOI:** 10.1186/s13068-021-01938-6

**Published:** 2021-04-08

**Authors:** Thitiwut Vongkampang, Krishnan Sreenivas, Jonathan Engvall, Carl Grey, Ed W. J. van Niel

**Affiliations:** 1 Division of Applied Microbiology, Lund University, P.O. Box 124, 221 00 Lund, Sweden; 2 Division of Biotechnology, Lund University, P.O. Box 124, 221 00 Lund, Sweden

**Keywords:** *Caldicellulosiruptor kronotskyensis*, Non-diauxic, Xylose uptake, Glucose uptake, Cellobiose uptake

## Abstract

**Background:**

*Caldicellulosiruptor kronotskyensis* has gained interest for its ability to grow on various lignocellulosic biomass. The aim of this study was to investigate the growth profiles of *C*. *kronotskyensis* in the presence of mixtures of glucose–xylose. Recently, we characterized a diauxic-like pattern for *C. saccharolyticus* on lignocellulosic sugar mixtures. In this study, we aimed to investigate further whether *C*. *kronotskyensis* has adapted to uptake glucose in the disaccharide form (cellobiose) rather than the monosaccharide (glucose).

**Results:**

Interestingly, growth of *C*. *kronotskyensis* on glucose and xylose mixtures did not display diauxic-like growth patterns. Closer investigation revealed that, in contrast to *C. saccharolyticus*, *C*. *kronotskyensis* does not possess a second uptake system for glucose. Both C. *saccharolyticus* and *C*. *kronotskyensis* share the characteristics of preferring xylose over glucose. Growth on xylose was twice as fast (*μ*_max_ = 0.57 h^−1^) as on glucose (*μ*_max_ = 0.28 h^−1^). A study of the sugar uptake was made with different glucose–xylose ratios to find a kinetic relationship between the two sugars for transport into the cell. High concentrations of glucose inhibited xylose uptake and vice versa. The inhibition constants were estimated to be *K*_I,glu_ = 0.01 cmol L^−1^ and *K*_I,xyl_ = 0.001 cmol L^−1^, hence glucose uptake was more severely inhibited by xylose uptake. Bioinformatics analysis could not exclude that *C*. *kronotskyensis* possesses more than one transporter for glucose. As a next step it was investigated whether glucose uptake by *C*. *kronotskyensis* improved in the form of cellobiose. Indeed, cellobiose is taken up faster than glucose; nevertheless, the growth rate on each sugar remained similar.

**Conclusions:**

*C*. *kronotskyensis* possesses a xylose transporter that might take up glucose at an inferior rate even in the absence of xylose. Alternatively, glucose can be taken up in the form of cellobiose, but growth performance is still inferior to growth on xylose. Therefore, we propose that the catabolism of *C*. *kronotskyensis* has adapted more strongly to pentose rather than hexose, thereby having obtained a specific survival edge in thermophilic lignocellulosic degradation communities.

## Background

Our reliance on fossil fuels resulted in massive greenhouse gases (GHGs) being released into the atmosphere. Biofuels, as an alternative, may contribute to the reduction of CO_2_ emissions, helping to keep the Paris Agreement that was determined to keep the global temperature below 2 $^\circ{\rm C}$ above the pre-industrial level [1]. Of the biofuels, biohydrogen is an interesting energy carrier due to that it does not emit carbon dioxide during combustion. In addition, hydrogen has a gravimetric energy content approximately of 122 kJ·g^-1^, which is threefold higher than carbon-based fuels [2].

Strictly anaerobic thermophilic bacteria of the genus of *Caldicellulosiruptor* are promising for biohydrogen production [3]. These Gram-positive bacteria grow optimally at a temperature of 70–75 $^\circ{\rm C}$. The most interesting feature is their ability to grow on a broad-spectrum of substrates, including poly-, oligo-, di- and, monosaccharides [4]. Among these *Caldicellulosiruptor* species, *C. saccharolyticus* is the most studied so far, including its physiology related to the sugar transporters [5]. *C. saccharolyticus* was isolated from thermal springs in New Zealand [6] and its completed genomic sequence has been annotated for its metabolism and transporter systems [7]. More recently, the whole genome of *C. saccharolyticus* was reannotated to improve the prediction of coding sequences [8]. *C. saccharolyticus* has been especially studied for its performance on sugar mixtures in lignocellulosic hydrolysates [9–11]. Interestingly, a recent study revealed that *C. saccharolyticus* displayed diauxic-like growth patterns on xylose–glucose mixtures [11].

In 2008, *Caldicellulosiruptor kronotskyensis* was isolated from thermal springs in Kamchatka, Russia. This species was considered akin to *C. saccharolyticus* and other species in the genus of *Caldicellulosiruptor*, with genome similarity levels between 94.8–97.7% [12], but the physiology of *C*. *kronotskyensis* has hardly been studied. During recent years, *C*. *kronotskyensis* has gained more interest due to its tāpirin proteins that attach to lignocellulosic materials [13] and it is well equipped for the breakdown of lignocellulosic biomass [14]. In addition, *C*. *kronotskyensis* was studied for its xylanase [15, 16], pectate lyase for the deconstruction of plant biomass [17] and has been used to enhance polyhydroxybutyrate (PHB) formation in a sequential fermentation [18]. Therefore, *C*. *kronotskyensis* is a potential platform for the production of biofuels and valuable chemicals.

Cellulose and hemicellulose are the major constituents of lignocellulosic biomass. Cellulose is a linear polymer consisting of glucose units linked by β-1,4-glycosidic bonds [19]. The hydrolysis of cellulose typically produces cellobiose and other oligomers. Hemicellulose is a heteropolymer with a side chain that comprises pentoses and hexoses, i.e., xylose, glucose, mannose, and other derivatives [20].

The current study focused on the physiology of *C*. *kronotskyensis* growing on mixtures of xylose and glucose such as in lignocellulosic hydrolysates. The growth profiles of *C*. *kronotskyensis* were evaluated against those of *C. saccharolyticus* in a former study [11]. Due to a difference in the growth pattern between these two species, the uptake of xylose and glucose in *C*. *kronotskyensis* was studied in further detail, which included also cellobiose as an alternative source of glucose. The generated data revealed that *C*. *kronotskyensis* might have adapted to xylose-based metabolism and takes up glucose preferentially, in the form of cellobiose and possibly other oligosaccharides, which may pinpoint its micro-niche in lignocellulosic degradation in its natural environment.

## Results

### Growth profiles on the single sugar

**Fig. 1:**
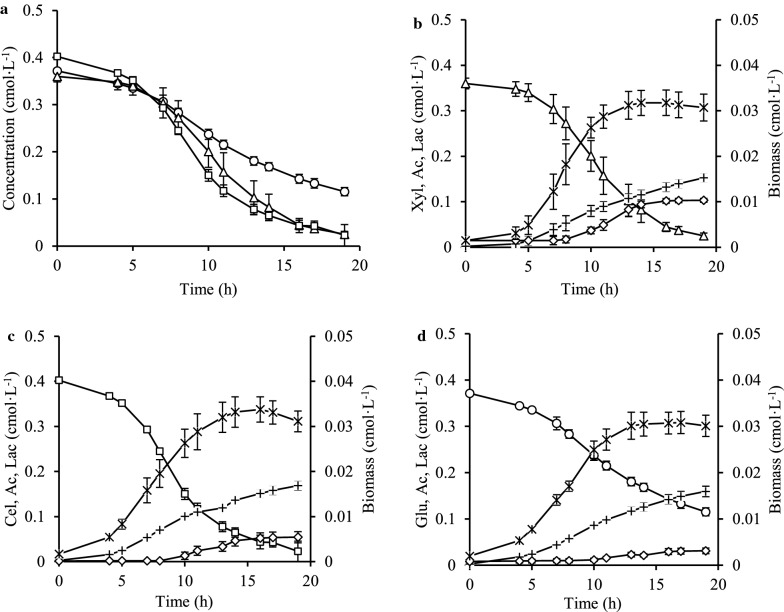
Growth pattern, sugar consumption and products formation on single sugar: **a** summary of sugar uptake profiles; **b** growth of *C*. *kronotskyensis* on 10 g·L^−1^ xylose (Case A); **c** growth of *C*. *kronotskyensis* on 10 g·L^−1^ cellobiose (Case B); **d** growth of *C*. *kronotskyensis* on 10 g·L^−1^ glucose (Case C). (Open triangle) xylose (Xyl); (open square) cellobiose (Cel); (open circle) glucose (Glu); ( multiplication) biomass; (open diamond) lactate (Lac); ( plus) acetate (Ac).

Batch cultivations of *C*. *kronotskyensis* on 10 g·L^−1^ of xylose (Case A), 10 g·L^−1^ of glucose (Case B), and 10 g·L^−1^ of cellobiose (Case C) were performed in a CSTR to monitor substrate utilization, biomass, and products formation. *C*. *kronotskyensis* assimilated xylose and cellobiose faster than glucose (Fig. 1a). However, there was no difference in biomass and acetate production during the fermentation of each sugar. For the fermentation on xylose, lactate production was twice and thrice higher than for the fermentation on cellobiose and glucose, respectively (Fig. 1b–d).

### Xylose–glucose uptake competition

**Fig. 2:**
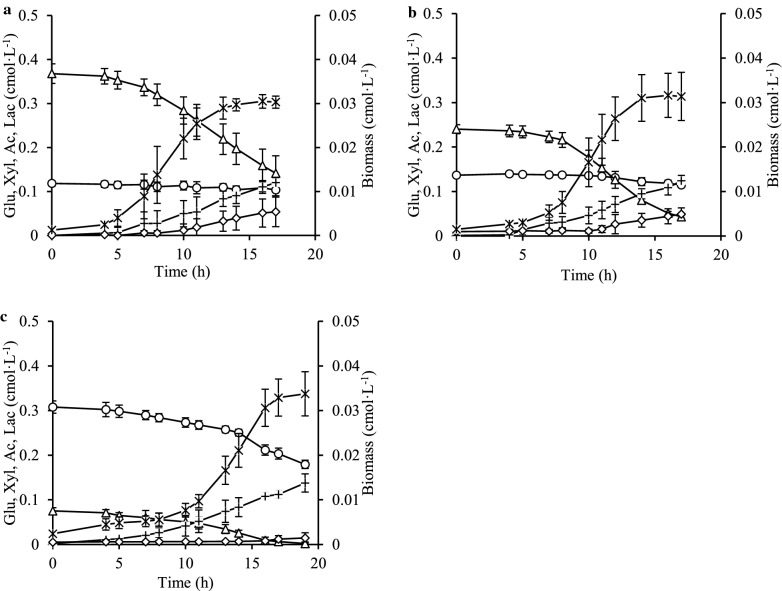
Growth pattern, sugar consumption and products formation for xylose–glucose competition: **a** growth of *C*. *kronotskyensis* on 12 g·L^−1^ xylose and 2 g·L^−1^ glucose (Case D); **b** growth of *C*. *kronotskyensis* on 9 g·L^−1^ xylose and 3 g·L^−1^ glucose (Case E); **c** growth of *C*. *kronotskyensis* on 3 g·L^−1^ xylose and 9 g·L^−1^ glucose (Case F). (Open triangle) xylose (Xyl); (open circle) glucose (Glu); ( multiplication) biomass; (open diamond) lactate (Lac); ( plus) acetate (Ac).

**Fig. 3:**
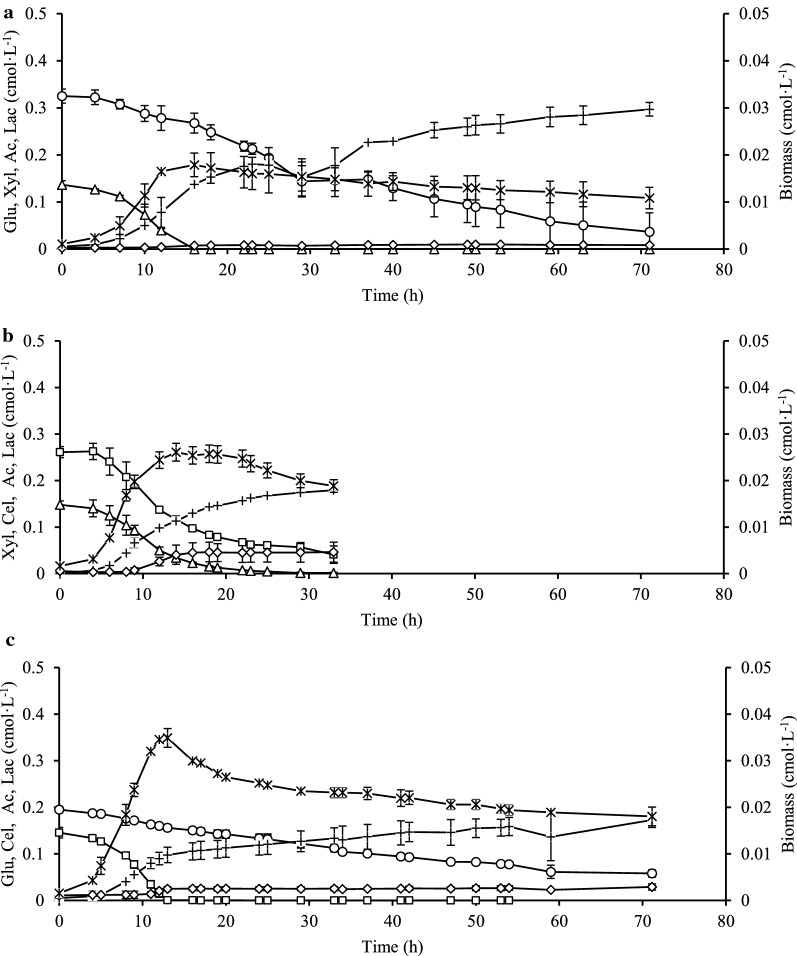
Growth pattern, sugar consumption and products formation on mixed sugars: **a** growth of *C*. *kronotskyensis* on 3.4 g·L^−1^ xylose and 7.3 g·L^−1^ glucose (Case D); **b** growth of *C*. *kronotskyensis* on 3.4 g·L^−1^ xylose and 7.3 g·L^−1^ cellobiose (Case E); **c** growth of *C*. *kronotskyensis* on 5 g·L^−1^ glucose and 5 g·L^−1^ cellobiose (Case F). (Open triangle) xylose (Xyl); (open circle) glucose (Glu); (open square) cellobiose (Cel); (multiplication) biomass; (open diamond) lactate (Lac); (plus) acetate (Ac).

*C*. *kronotskyensis* was cultivated in media containing xylose and glucose mixtures with different ratios (Table 1, Cases D–F). The results of Case D and Case E revealed that xylose was consumed from the early logarithmic phase until the stationary phase. On the other hand, *C*. *kronotskyensis* did hardly take up glucose when the amount of xylose was higher than 0.1 cmol·L^−1^ (Fig. 2a, b). Moreover, the uptake of glucose (Case E) began only after the level of xylose became below 0.05 cmol·L^−1^ (Fig. 2b). This phenomenon was also observed in Case F (Fig. 2c) and Case G (Fig. 3a). Cultures containing higher concentrations of xylose in the sugar mixtures produced more lactate, i.e., lactate formation in Case D and Case E were approximately thrice and a half higher, respectively, than in Case F (Fig. 2). However, there was no difference in acetate production and biomass formation among these three cases.

**Table 1:** Fermentation conditions performed in this study

Name	Cultivation conditions
Case A	10 g·L^−1^ xylose
Case B	10 g·L^−1^ cellobiose
Case C	10 g·L^−1^ glucose
Case D	2 g·L^−1^ glucose and 12 g·L^−1^ xylose
Case E	3 g·L^−1^ glucose and 9 g·L^−1^ xylose
Case F	9 g·L^−1^ glucose and 3 g·L^−1^ xylose
Case G	7.3 g·L^−1^ glucose and 3.4 g·L^−1^ xylose
Case H	7.3 g·L^−1^ cellobiose and 3.4 g·L^−1^ xylose
Case I	5 g·L^−1^ cellobiose and 5 g·L^−1^ glucose

### Growth profiles on the mixed sugars

In Case G, xylose depleted earlier than glucose. Subsequently, glucose was not taken up faster as was expected but continued at a constant speed (Fig. 3a). For Case H, glucose was substituted by cellobiose, which resulted in that both sugars were concurrently consumed (Fig. 3b). Surprisingly, the total fermentation period (Case H) was approximately half the time shorter comparing to the other two cases where glucose was presented (Cases G and I). For Case I (Fig. 3c), the uptake profile of cellobiose was similar to that of the xylose uptake profile in Case G and Case H, but the uptake profile of glucose was quite similar to the glucose profile in Case G.

**Fig. 4:**
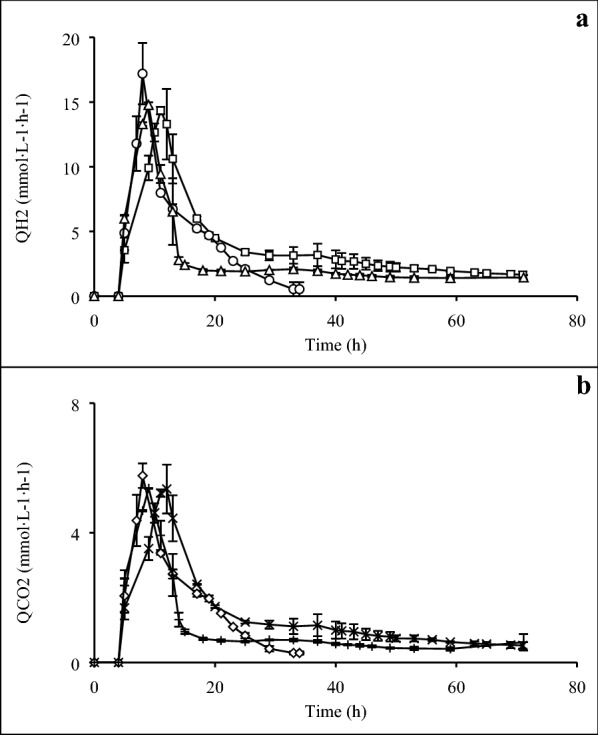
Volumetric hydrogen productivity (Q_H2_) and volumetric carbon dioxide productivity (Q_CO2_). **a** Q_H2_ obtained in Case G (open square), Case H (open circle), and Case I (open triangle); **b** Q_CO2_ obtained in Case G ( multiplication), Case H (open diamond), and Case I ( plus)

Acetate was the main soluble by-product obtained from the growth profiles during the fermentation of mixed sugars. Acetate formation in Case G was approximately 1.5-fold higher than the acetate level in both Case H and Case I. Lactate in Case H was approximately 1.5 times higher than in Case I and just about fivefold higher than in Case G. Biomass observed in Case I was 0.035 cmol·L^−1^, which was one and a half-fold greater than Case H (0.026 cmol·L^−1^) and almost twice higher for Case G (0.018 cmol·L^−1^) (Fig. 3a–c). Neither of the monitored profiles of Q_H2_ and Q_CO2_ during the fermentation of mixed sugars displayed any diauxic-like growth patterns (Fig. 4a, b), as it has been observed for *C. saccharolyticus* [11].

### Estimation of ***µ***_max_ from the fermentations on single sugar

The *µ*_max_ of the culture of *C*. *kronotskyensis* on xylose (Case A) was 0.57 h^−1^, which was nearly twice higher than the *µ*_max_ of the culture on cellobiose (Case B) and glucose (Case C) (Table 2). For the xylose–glucose competition, the *µ*_max_ was higher at higher xylose/glucose ratio (Cases D and E) than at a lower ratio. With a mixture of cellobiose and xylose (Case H), the culture reached a *µ*_max_ between the *µ*_max_ of each sugar alone and was significantly higher than for the mixture of xylose–glucose (Case G) and cellobiose–glucose (Case I).

**Table 2:** Calculated yields, carbon balance, redox balance, and µ_max_ of the fermentations in this study

Sugar	Yield					Carbon balance	Redox balance	*µ*_max_ (h^−1^)
	*Y*_SH_	*Y*_SC_	*Y*_SA_	*Y*_SL_	*Y*_SX_			
	[mol·cmol^−1^]		[cmol·cmol^−1^]					
Case A	0.36 ± 0.02	0.14 ± 0.01	0.46 ± 0.02	0.26 ± 0.04	0.19 ± 0.02	1.03 ± 0.03	1.05 ± 0.04	0.57
Case B	0.35 ± 0.05	0.14 ± 0.02	0.45 ± 0.05	0.14 ± 0.01	0.15 ± 0.01	1.08 ± 0	1.09 ± 0	0.30
Case C	0.50 ± 0.02	0.20 ± 0.01	0.61 ± 0.02	0.09 ± 0.04	0.17 ± 0.01	1.04 ± 0.02	1.06 ± 0.03	0.28
Case D	0.42 ± 0.08	0.16 ± 0.03	0.52 ± 0.10	0.22 ± 0.11	0.09 ± 0.06	1.07 ± 0.02	1.03 ± 0.02	0.43
Case E	0.50 ± 0.01	0.19 ± 0.01	0.57 ± 0.08	0.18 ± 0.06	0.25 ± 0.08	1.04 ± 0.07	0.97 ± 0.04	0.43
Case F	0.54 ± 0.03	0.20 ± 0.01	0.67 ± 0.13	0.05 ± 0.05	0.15 ± 0.07	1.04 ± 0.06	1.01 ± 0.03	0.21
Case G	0.38 ± 0.03	0.15 ± 0.01	0.48 ± 0.04	0.14 ± 0.05	0.14 ± 0.01	1.12 ± 0.05	1.06 ± 0.04	0.25
Case H	0.54 ± 0.01	0.21 ± 0.01	0.70 ± 0.02	0.02 ± 0	0.11 ± 0	1.11 ± 0.02	1.08 ± 0.01	0.41
Case I	0.50 ± 0.06	0.20 ± 0.02	0.69 ± 0.08	0.16 ± 0.12	0.26 ± 0.04	0.99 ± 0.01	0.95 ± 0.01	0.31

### Determination of yields, carbon balance and redox balance

In this study, the carbon balance indicated good carbon recovery, and, all redox balance represented above 95%, denoting reliable H_2_ measurements. Yields were calculated by using the overall product formed divided by the total substrate converted. For single sugar, the yield of hydrogen (*Y*_SH_), carbon dioxide (*Y*_SC_), and acetate (*Y*_SA_) for glucose alone (Case C) were higher than for xylose (Case A) and cellobiose (Case B). In contrast, the yield of lactate (*Y*_SL_) for Case A was greater than Case B and Case C. Moreover, the yield of biomass (*Y*_SX_) of these cases (Cases A–C) was at a similar level (Table 2).

The yield of hydrogen (*Y*_SH_), carbon dioxide (*Y*_SC_), and acetate (*Y*_SA_) for the different xylose–glucose ratios (Cases D–F) were overall quite similar. Except for the yield of lactate (*Y*_SL_) for Case F was slightly lower than for Case D and Case E. The highest yield of biomass (*Y*_SX_) was in Case E, followed by the *Y*_SX_ in Case F, which was similar to the *Y*_SX_ in Case D (Table 2).

For the cultivation of the mixed sugars, the yield of hydrogen (*Y*_SH_), carbon dioxide (*Y*_SC_), and acetate (*Y*_*SA*_) in Case H and Case I were similar, but both were significantly higher than Case G. However, the yield of lactate (*Y*_SL_) in Case H was lower than Case G and Case I. Furthermore, Case I showed a higher yield of biomass (*Y*_SH_) than Case G and Case H.

### Specific substrate consumption rate (qS) and specific H_2_ production rate (qH_2_)

**Fig. 5:**
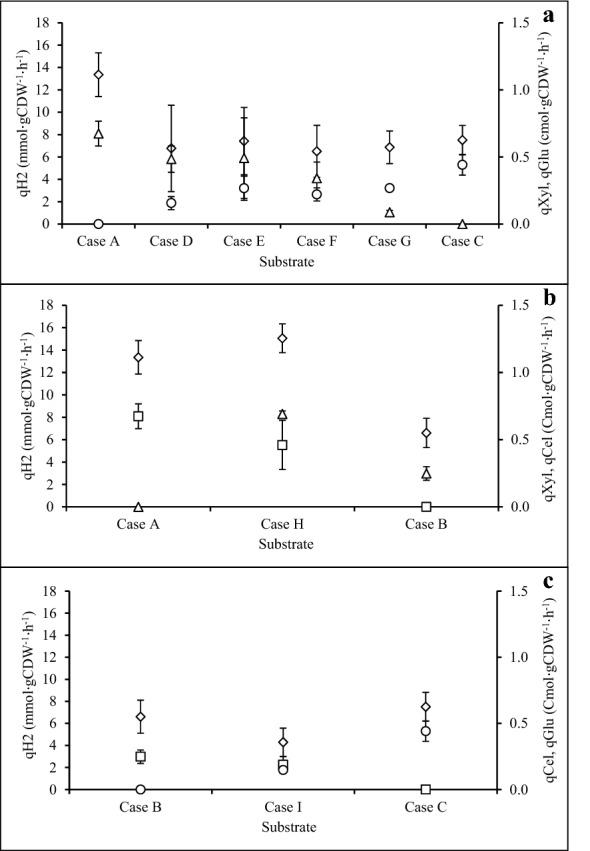
Specific substrate consumption (qS) and specific hydrogen production (qH_2_). **a** Specific xylose consumption (qXyl), specific glucose consumption (qGlu), and specific hydrogen production (qH_2_) for Case D, Case E, Case G, and Case F comparing with Case A and Case C. **b** Specific xylose consumption (qXyl), specific cellobiose consumption (qCel), and specific hydrogen production (qH_2_) for Case H comparing with Case A and Case B. **c** Specific xylose consumption (qXyl), specific cellobiose consumption (qCel), and specific hydrogen production (qH_2_) for Case I comparing with Case B and Case C. (Open triangle) qXyl; (open square) qCel; (open circle) qGlu; (open diamond) qH_2_

The specific substrate consumption (qS) on single sugar and mixed sugars were calculated based on substrate in cmol·L^−1^·h^−1^ divided by cell dry weight (gCDW·L^−1^). The specific H_2_ production (qH_2_) of each fermentation was calculated from the volumetric hydrogen productivity (Q_H2_, mmol·L^−1^·h^−1^) divided by cell dry weight (gCDW·L^−1^). For Case A, the specific xylose consumption (qXyl) was approximately 0.67 cmol·gCDW^−1^·h^−1^, whereas the specific glucose consumption (qGlu) in Case C was 0.44 cmol·gCDW^−1^·h^−1^ (Fig. 5a). During the fermentation with different xylose–glucose mixtures, qXyl decreased with decreasing xylose/glucose ratios. A similar phenomenon also occurred in case of qGlu, which decreased with increasing xylose/glucose ratios. It is worth noting that the qH_2_ of culture fermenting xylose was significantly decreased from 13 to 6.8 mmol·gCDW^−1^·h^−1^ when glucose was present. For the mixture of xylose and cellobiose (Case H), the specific cellobiose consumption (qCel) was lower than both qXyl and qGlu (Fig. 5b). Interestingly, qCel increased dramatically from 0.25 to 0.7 cmol·gCDW^−1^·h^−1^ in the case of xylose–cellobiose mixture (Case H) while qXyl showed no significant decrease. The qH_2_ of Case H improved somewhat when compared with qH_2_ of Case B. For Case I, only qGlu considerably decreased from 0.44 to 0.15 cmol·gCDW^−1^·h^−1^, whereas qCel did not show any remarkably change (Fig. 5c). Moreover, there were no differences between the qH_2_ obtained in Case B, Case C, and Case I.

### Inhibition kinetics of xylose and glucose uptake

As Fig. 5a depicts, the specific consumption rate of both xylose and glucose declined in the sugar mixture when more of the other sugar was present. This indicated competitive inhibition and, therefore, the inhibition constant (*K*_*I*_) for xylose (*K*_I,xyl_) and glucose (*K*_I,glu_) were estimated (Eqs. 3,4) focusing only on the cases of the xylose–glucose mixture (Cases D–G). It was found that the *K*_I,glu_ was 0.01 cmol·L^−1^, being ten times higher than *K*_I,xyl_ (0.001 cmol·L^−1^), or glucose uptake was more severely inhibited by xylose than vice versa.

### Competitive of sugar uptake in *C*.* kronotskyensis*

**Fig. 6:**
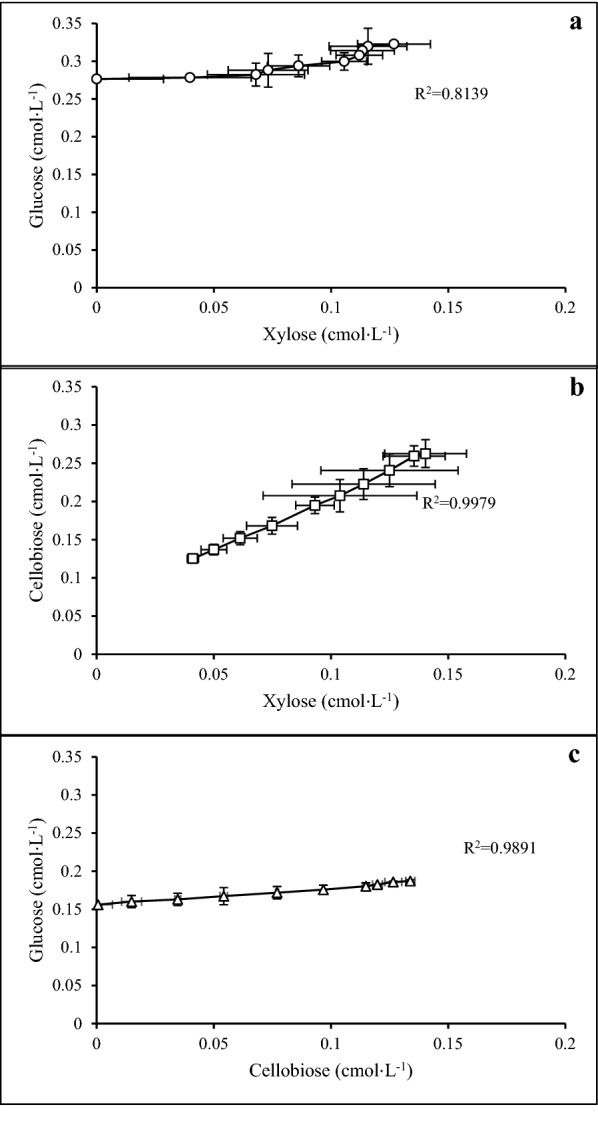
Stoichiometry of substrate uptake in the cultures on mixed sugars during the logarithmic phase. **a** Relation between xylose and glucose uptake. **b** Relation between xylose and cellobiose uptake. **c** Relation between cellobiose and glucose uptake. (Open circle) Case G; (open square) Case H; (open triangle) Case I

The sugars in Case G, Case H, and Case I were plotted to exhibit the relative stoichiometry of each sugar uptake during the exponential phase. As depicted in Fig. 6a, the uptake of xylose and glucose (Case G) was seemingly linear depicting a certain metabolic stoichiometry (R^2^= 0.814).Similarly, sugar uptake of the other two cases (Cases H and I) was linearly related throughout the cultivation (R^2^ ≥ 0.989)  (Fig. 6b, c) reflecting a more stable stoichiometry than in Case G, which might be related to the use of two separate sugar transporters.

### Bioinformatics related on sugar transporters

Since carbohydrate transporters play a crucial role in sugar uptake during the growth of bacteria, obvious differences in sugar transporters between the already studied C. saccharolyticus [21] and C. kronotskyensis were compared using genome alignment produced by Mauve. Upon analysis of the genome alignment, there were two sugar transporters that were absent in *C. kronotskyensis*. These sugar transporters belong to the Dpp/Opp family for fructose and sucrose uptake as well as a CUT1 family sugar transporter for xyloglucan (Table 3).

**Table 3:** Comparison of carbohydrate transporters between *C*. *kronotskyensis* (the current study) and *C. saccharolyticus *

ABC transporter (Csac_)	Group	ABC transporter (Calkro_)	Features
0238,0240–0242	CUT2	0382,0384–0386	Arabinose, galactose, xylose
0261–0265	Dpp/Opp	None	Fructose, sucrose
0427–0428,0431	CUT1	0283–0284,0287	Maltodextrin
0440–0442	CUT1	2234–2236	Galactose
0692–0694	CUT1	2010–2012	Monosaccharides
1028–1032	Dpp/Opp	0798–0802	Monosaccharides
1557–1559	CUT1	None	Xyloglucan
2321–2322,2324,2326	CUT1	0930,0932,0933–0934	Glucose, xylose, fructose
2412–2414	CUT1	2389–2391	Xylooligosaccharides
2417–2419	CUT1	2394–2396	Xylooligosaccharides
2491–2493	CUT1	0321–0323	Xylose, glucose, fructose
2504–2506	CUT2	0128–0130	Xylose, glucose, fructose
2514–2516	CUT1	0108–0110	Glucooligosaccharides

The ABC transporters (Csac_), group and features of *C. saccharolyticus* were taken from a previous study [21]

## Discussion

This study was performed after the observation that *C*. *kronotskyensis* did not possess a diauxic-like growth pattern on xylose–glucose fermentations (Fig. 4) as has been described for *C. saccharolyticus* in a previous study [11]. Both volumetric productivity profiles of hydrogen (Q_H2_) and carbon dioxide (Q_CO2_) consist of only one peak corresponding to the exponential growth phase. In contrast to *C. saccharolyticus*, there is no second peak after xylose has been consumed. Instead, Q_H2_ and Q_CO2_ continued to decline even though plenty of glucose was still present. In the presence of xylose in both species a transporter is expressed that takes up both xylose and glucose. The mechanism behind the diauxic-like growth pattern in *C. saccharolyticus* was based on a second transporter for glucose being expressed after depletion of xylose [11]. *C*. *kronotskyensis* has a genetic similarity of approximately 94% with *C. saccharolyticus* [12], and one of the differences is a lower number of ABC transporters for sugar uptake in *C*. *kronotskyensis* (Table 3). The results obtained in this study indicated that *C*. *kronotskyensis* might be missing a second transporter for glucose. Based on this evidence, we studied the simultaneous uptake of glucose and xylose in *C*. *kronotskyensis* by means of one transporter using different concentration ratios of xylose and glucose. Like for *C. saccharolyticus* [11, 21], this transporter has a preference for xylose and at high concentrations of this sugar, whereas glucose is hardly taken up. For obvious reasons, a competitive inhibition was assumed, and the kinetic analysis demonstrated that xylose inhibited glucose uptake more severely than the other way around. The uptake of both sugars simultaneously affected the overall growth rate, but there is no clear indication that it affected the biomass yield and hydrogen yield (Table 2).

Due to a low preference for glucose, it could be expected that *C*. *kronotskyensis* is taking up this hexose preferably in the form of oligosaccharides. Several oligosaccharide transporters can be assigned in the genome of this species (Table 3), and indeed cellobiose was taken up via a separate transporter in the presence of xylose according to the concentration profiles of the two sugars in Fig. 3b. Both sugars were taken up simultaneously with a stable stoichiometry as demonstrated in Fig. 6b. A similar relationship was observed for the glucose and cellobiose mixture (Fig. 6c). Cellobiose might be taken up through the transporters labelled as Calkro_0108-0110, as being predicted for gluco-oligosaccharides [13]. However, the non-linear correlation between simultaneous uptake of xylose and glucose is connected to the competition taking place at the xylose transporter (Fig. 6a). Curiously, after depletion of cellobiose in Case I, the uptake of glucose continued with the same slow uptake throughout the remainder of the fermentation. The fact that uptake of glucose was linear probably due to that was no further increase of cell mass, which might indicate either limitation of another nutrient or is caused by a yet unknown mechanism. *C. saccharolyticus*, *C. owensensis*, and *C. kristjanssonii* have in common that they prefer xylose over other sugars in mixed substrate cultures. Nonetheless, when these three species were cultivated together, the uptake of glucose and xylose occurred concurrently [22]. This sugar preference pinpoints to an adaptation to a specific niche within the thermophilic lignocellulosic degradation community. Other such specialization within lignocellulosic degradation communities has been indicated before [23].

With xylose, the *µ*_max_ was 0.57 h^−1^ which is significantly higher than the *µ*_max_ observed for *C. saccharolyticus* (0.25 h^−1^) [24] and *C. owensensis* (0.20 h^−1^) [25]. In the current study, cellobiose uptake was also superior to that of glucose even though the *µ*_max_ on each of these sugars was quite similar. The *µ*_max_ on glucose obtained by *C*. *kronotskyensis* (0.28 h^−1^) was slightly higher than the *µ*_max_ obtained in *C. saccharolyticus* (0.23 h^−1^) [11, 22]. In the case of glucose and xylose mixtures, the µ_max_ increased with the xylose/glucose ratio as the growth rate was mainly determined by xylose but inhibited by glucose. This phenomenon was further investigated by a competitive kinetic model that indicated that glucose was more severely inhibited by xylose than vice versa. Interestingly, the µ_max_ of cultures on xylose and cellobiose mixtures was halfway between the maximum specific growth rates for each case on single sugar (Table 2), which is correlated with a certain conversion stoichiometry between cellobiose and xylose (Fig. 6b).

The qXyl was not significantly different in the presence of cellobiose in the medium. As aforementioned, xylose and cellobiose were consumed concurrently. Interestingly, qCel obtained in Case H considerably improved almost threefold compared to qCel in Case B (Fig. 5b). Therefore, both qXyl and qCel also confirmed that the assimilation of xylose and cellobiose in *C*. *kronotskyensis* occurred with different sugar transporters.

## Conclusion

The current study highlighted the lack of a diauxic-like growth pattern in *C*. *kronotskyensis* during fermentation of the xylose–glucose mixture. *C*. *kronotskyensis* has the highest preference for xylose and cellobiose compared to glucose. This is exemplified by the lack of a specific glucose transporter and the highest growth rate observed with xylose in single-sugar cultures. Nevertheless, the highest Q_H2_ has been observed in the presence of glucose and/or cellobiose. This study further supports that members of the genus *Caldicellulosiruptor* have adapted to xylose and disaccharides (cellobiose) rather than glucose as primary substrates, which may give them a competitive edge in thermophilic lignocellulosic degradation. It further shows that *C*. *kronotskyensis* is a promising candidate for biohydrogen production from lignocellulosic material.

## Materials and methods

### Microorganism and cultivation medium

*Caldicellulosiruptor kronotskyensis* DSM 18902 was purchased from the Deutsche Sammlung von Mikroorganismen und Zellkulturen (Braunschweig, Germany). The inoculum was prepared by subculturing routinely in 250-mL serum flasks containing 50 mL of modified DSM medium 640. The medium composition used in this study was previously described by [25]. The cultivation medium (DSM medium 640) without yeast extract, 1000X vitamin solution, and modified SL-10 solution were prepared according to [25, 26].

### Experimental design

The batch cultivations were performed with different sugar ratios (Table 1). Briefly, the control conditions were Cases A, B, and C, which were single-sugar cultivation on xylose, cellobiose, and glucose, respectively. Uptake kinetics on different sugar ratios were performed in Cases D, E, F, and G. The sugar mixtures in Case G were based on the ratio seen in wheat straw hydrolysate [11]. In addition, each of these sugars was combined with cellobiose to determine the preference of sugar uptake (Cases H and I). All cultivations were done in biological duplicate.

### Fermentation setup

Batch fermentations were performed in a 3-L bioreactor provided with ADI 1025 Bio-console and ADI 1010 Bio-Controller (Applikon, Schiedam, The Netherlands) at a working volume of 1 L. The medium was stirred at 250 rpm and kept at pH 6.9 ± 0.1 with automatic titration with 4 M NaOH. The medium was continuously stripped with N_2_ gas containing less than 5 ppm O_2_ (AGA Gas AB, Sweden) at a rate of 100 mL·min^-1^. For xylose, the sparging gas was turned off after inoculation for 4 h to allow accumulation of CO_2_ as previously reported [26], which is necessary to promote initial growth. Then, N_2_ gas was continuously sparged again throughout cultivation. 5 mL of each culture was taken at specific intervals and spun down to collect supernatant and stored at − 20 $^\circ{\rm C}$ until further analysis.

### Analytical methods

Gas samples were taken from the bioreactor’s headspace to quantify hydrogen gas and carbon dioxide with gas chromatography using an Agilent 7890B (Santa Clara, CA, USA) equipped with a TCD and a ShinCarbon ST 50/80 UM (2 m × 1/16 × 1 mm) column. Helium gas was fed through the system with a flow rate of 10 mL·min^−1^ at 80 $^\circ{\rm C}$ for 1 min. The temperature was increased to 100 $^\circ{\rm C}$ and held for 4 min followed by a temperature ramp to 160 $^\circ{\rm C}$ for 2 min. The built-in software obtained from Agilent was used for analysis and calculating the percentages of H_2_ and CO_2_. The calibration curve of H_2_ and CO_2_ were validated after setting up of the GC and used for analysis afterwards.

Glucose, xylose, and cellobiose were detected on a Shodex SP-0810 column (8 × 300 mm, Shodex, Japan) using Milli-Q water as a mobile phase at a flow rate of 0.6 mL·min^−1^ at 60 $^\circ{\rm C}$. Acetate, lactate, propionate, and ethanol were quantified by high-performance liquid chromatography (HPLC; Waters, Milford, MA, USA) equipped with an Aminex HPX-87H ion-exchange column (7.8 × 300 mm, Bio-Rad, Hercules, USA). The column was operated at 60 $^\circ{\rm C}$ with 5 mM H_2_SO_4_ as a mobile phase at a flow rate of 0.6 mL·min^−1^.

### Biomass concentration

An Ultrospec 2100 pro UV/visible spectrophotometer (Amersham Biosciences, UK) was used to determine the optical density (OD) of the culture at 620 nm. A culture volume of 10 mL was filtrated through a pre-weight Supor-200, 0.2-µm filter (PALL Life Sciences, Mexico) to estimate cell dry weight (CDW). The filters were rinsed thrice with 5 mL of Milli-Q water and dried in a desiccator overnight. The dry filters were measured on an analytical balance (AG204 DeltaRange, Mettler Toledo, Ohio, USA). The CDW samples were done in duplicate. The correlation between CDW and OD_620_ was calculated to estimate CDW of each sample according to:

1 $$\mathrm{CDW}=a\left[\mathrm{OD}\right]+b,$$

where CDW and *b* are cell dry weight and linear regression constant (g·L^−1^), respectively. *a* is a slope of the linear regression. The values for *a* and *b* varied with the sugar (combinations) used in the cultures. The equation was obtained as described per [25].

### Mathematical modeling to predict µ_max_

The modified Gompertz equation as per Zwietering et al. [27] and optical density (OD) obtained from each case were used for the estimation of *µ*_max_:

2 $$y=A\mathrm{exp}\{-\mathrm{exp}[\frac{{\mu }_{m}\mathrm{exp}}{A}\left(\lambda -t\right)+1]\},$$

where *A* is a term of the asymptotic value, µ_*m*_ is the specific growth rate (*µ*_max_, h^−1^) and *λ* is the length of the lag phase (*h*).

### Inhibition constant (***K***_***I***_) of glucose and xylose

The inhibition of glucose uptake by xylose and vice versa is assumed to be based on competitive inhibition of the substrate-binding protein of the ABC transporter. The two equations that can be derived are:

3 $${V}_{\mathrm{xyl}}=\frac{{V}_{m,\mathrm{ xyl }\cdot }\frac{X}{{\mathrm{K}}_{S,\mathrm{xyl}}}}{1+\frac{X}{{K}_{\mathrm{S},\mathrm{xyl}}}+\frac{G}{{K}_{\mathrm{I},\mathrm{glu}}}},$$

and

4 $${V}_{\mathrm{glu}}=\frac{{V}_{m,\mathrm{glu }\cdot }\frac{G}{{\mathrm{K}}_{S,\mathrm{glu}}}}{1+\frac{G}{{K}_{S,\mathrm{glu}}}+\frac{X}{{K}_{I,\mathrm{xyl}}}},$$

where ${V}_{\mathrm{xyl}}$ and ${V}_{\mathrm{glu}}$ are xylose uptake rate and glucose uptake rate (cmol·L^−1^), respectively, ${V}_{\mathrm{m},\mathrm{xyl}}$ and ${V}_{\mathrm{m},\mathrm{glu}}$ are the maximum substrate uptake rate (cmol·L^−1^). The parameters $X$ and $G$ are the concentration of xylose and glucose (cmol·L^−1^), respectively.

The aim was to obtain a good appraisal of the inhibition constants for the uptake of xylose (*K*_*I*,glu_) and glucose (*K*_*I*,xyl_) in the cultures of sugar mixtures (Cases D–E). To minimize the number of unknown parameter values, we assumed that the *K*_*S*_ value of glucose (*K*_*S*,glu_) and xylose (*K*_S,xyl_) obtained for *C. saccharolyticus* from the previous study [11] was similar to those of *C*. *kronotskyensis*: the *K*_*S*,glu_ and *K*_*S*,xyl_ being 0.01 and 0.0002 cmol·L^−1^, respectively. This assumption is adequate as both transporters displayed the same profile of xylose and glucose uptake and 10-20% change in the *K*_*S*_-value had less than 5% influence on the values of *K*_*I*,glu_ and *K*_*I*,xyl_.

### Bioinformatics study related to sugar transporter

The sugar transporters of *C*. *kronotskyensis* related in this study were investigated and compared with those sugar transporters identified in *C. saccharolyticus* in a previous study [21]. The completed DNA sequences and gene encoding for transporters of *C*. *kronotskyensis* (https://www.ncbi.nlm.nih.gov/nuccore/NC014720) and *C. saccharolyticus* (https://www.ncbi.nlm.nih.gov/nuccore/ CP000679) are available at the National Center for Biotechnology Information (NCBI; Bethesda, MD, USA). The completed genome sequences of these two species were compared by using Mauve program (Darling lab, University of Technology Sydney).

## Data Availability

The datasets during and/or analyzed during the current study are available from the corresponding author on reasonable request.
